# Incidence and mortality of new-onset glucose disorders in peritoneal dialysis patients in China: a meta-analysis

**DOI:** 10.1186/s12882-020-01820-x

**Published:** 2020-04-29

**Authors:** Yanan Shi, Jiajie Cai, Chunxia Shi, Conghui Liu, Zhongxin Li

**Affiliations:** Department of Nephrology, Beijing LuHe Hospital, Capital Medical University, No. 82 Xinhua South Road, Tongzhou District, Beijing, 101100 China

**Keywords:** Diabetes mellitus, Peritoneal dialysis, Glucose metabolism disturbances, Meta-analysis

## Abstract

**Background:**

Dialysis patients are at high risk of developing glucose metabolism disturbances (GMDs), such as diabetes mellitus (DM), impaired fast glucose (IFG), and impaired glucose tolerance (IGT). However, it is unclear about the incidence of GMDs in Chinese patients with peritoneal dialysis (PD), as well as the influence of new-onset DM (NODM) on the prognosis of PD patients. Therefore, we conducted this meta-analysis to address these issues.

**Methods:**

A comprehensive literature search was conducted using PubMed, Embase, Web of Science, SinoMed, and CNKI database for studies that evaluated the incidence of GMDs and mortality in patients with PD. Results were expressed as hazard ratio (HR), risk ratio (RR), or estimate (ES) with 95% confidence intervals (95%CIs).Meta-analysis was performed using a fixed-effects or random-effects model to pool the estimate.

**Results:**

Fifteen studies met the inclusion criteria and were included in this meta-analysis. Pooled results showed that, the incidences of NODM, NOIGT, and NOIFG were 12% (95%CI: 9, 15%; *P* < 0.001), 17% (95%CI: 4, 10%; P < 0.001) and 32% (95%CI: 3, 30%, P < 0.001), respectively. Compared with patients without NODM, PD patients with NODM had an increased risk of mortality (HR = 1.59, 95%CI: 1.28, 1.98; *P* < 0.001). There was no significant difference in the incidence of NODM between PD and hemodialysis (HD) patients (RR = 1.23, 95%CI: 0.61, 2.51; *P* = 0.562).

**Conclusion:**

Dialysis patients in China had an increased risk of developing GMDs, however, the dialysis modality did not have any significant impact on the incidence of NODM. NODM increased the mortality risk in patients undergoing PD. Thus, physicians should pay attention to the plasma glucose level in patients undergoing dialysis.

## Background

New-onset diabetes mellitus (NODM), impaired fast glucose (IFG), and impaired glucose tolerance (IGT) are common complications in chronic kidney disease (CKD) patients undergoing peritoneal dialysis (PD) or hemodialysis (HD) [1–3]. They have been demonstrated to increase the risk of cardiovascular events and death in general population [4, 5], especially in patients receiving dialysis [1–3]. Dialysis patients with diabetes have a poorer prognosis than those without diabetes [6].

Insulin resistance and concurrent hyperinsulinemia may occur in all the stages of CKD patients, even in patients with normal glomerular filtration rate, irrespective of the type of renal disease [7–9]. The dialysis therapy can partially improve insulin resistance, but not to the normal level [10]. Since glucose is used as the osmotic agents, hyperglycemia is commonly seen in PD patient [11]. This would result in worse survival [11].

Many studies have investigated the association between pre-existing DM at the initiation of dialysis and poor survival outcomes among CKD patients who underwent dialysis [12, 13]. However, few studies have reported the prevalence of NODM and its association with survival outcomes in dialysis patients [2, 11, 14]. The incidence of NODM varied greatly across different countries, with 12.7% at 2 years in HD patients in US [14], 4% at 1 year and 21% in 9 years in Taiwan [1]. Woodward et al. [14] collected data from the US Renal Data System, and reported that the prevalence of NODM to be 6% per year in dialysis patients. Whereas, in Asia, a high incidence of hyperglycemia has been observed in Chinese patients in Hong Kong, with a daily exchange of 1.5% glucose dialysate [11]. The incidence of NODM might be overestimated because the competing events were not taken into consideration in the analysis [15]. Moreover, the prevalence of NODM is different across patients undergoing HD or PD. CKD patients who underwent PD are usually younger than those undergoing HD [16–18], and HD patients may have higher risk of developing NODM than PD patients.

The worldwide number of CKD patients who underwent dialysis has been increased in the past decades. Previous studies have revealed that the incidence and prevalence of CKD patients undergoing dialysis are high in Hong Kong and Taiwan, China [11, 19]. Thus, we conducted this meta-analysis to investigate the incidence of GMDs in Chinese dialysis patients, to explore whether dialysis modality is associated with NODM, as well as whether NODM increases the risk of mortality.

## Methods

### Search strategy

We performed this meta-analysis in according to the methods of the *Cochrane Handbook for Systematic Reviews of Interventions* [20]. All the results were presented in accordance with the Preferred reporting Items for Systematic Reviews and Meta-Analyses (PRISMA) statement [21]. Scientific articles published in English or Chinese were searched from Pubmed, Embase, Web of Science, SinoMed (Chinese BioMedical Literature Service System, China), and CNKI (National Knowledge Infrastructure, China). Different retrieval formulas were established according to individual databases. The keywords we used were listed as followings: (diabetes OR new-onset diabetes OR de novo diabetes OR GMD OR IFG OR oral glucose tolerance test OR IGT) AND (dialysis OR PD OR hemodialysis)

.. The bibliographies of the previous reviews and of the included studies were also manually searched to identify other potentially eligible trials.

### Study inclusion and exclusion criteria

All studies published before January 5, 2020, in English or Chinese language, were considered for inclusion. To be included, studies must meet the following inclusion criteria: (1) study population: dialysis patients living in China; (2) study design: observational study, cohort study, or case-control studies; (3) outcome: incidence of NOGMD, including NODM, IGT and IFG, survival outcomes in PD patients. Studies were excluded from the final analysis if they did not focus on Chinese patients, or did not provide data of our interest, or published with the following types of study design: reviews, case reports, or policy analysis.

### Data extraction

Data extraction was performed by two independent investigators. A standardized Excel file was used to extract the following information from each of the included studies: first author’s name, year of publication, study design, sample size, patients’ baseline characteristics (age, gender, body mass index, and comorbidity), and outcomes. When several publications were from the same trial or population, we only included the latest or most information study in order to avoid the duplication of information.

### Methodological quality assessment

The methodological quality of each included study was assessed using the modified Newcastle-Ottawa (NOS) scale by two independent investigators [22]. The quality score of each study was given in according to the following three items, patient selection, comparability of the intervention/ control group, and outcome assessment [22]. The total score was 9 points, and higher scores indicated better quality. A study with a NOS scale greater than 5 points was considered to be high quality.

### Statistical analysis

Before the data were synthesized, heterogeneity was tested by using the Cochrane Q chi-square and *I*^2^statistic, in which *P* value < 0.1 or *I*^2^ > 50% were defined to have heterogeneity [20]. When substantial heterogeneity was identified, a randomized-effects model (DerSimonian-Laird method) [23] was used; otherwise, a fixed-effects model (Mantel-Haenszelmethod) [24] was preferred to pool the incidence rate and survival rate, as well as the 95% confidence intervals (95%CIs). The incidence rate was calculated with estimate (ES) and 95%CIs; survival rate was a time-to-event variable, thus it was expressed as hazard ratio (HR) with 95%CIs; the comparison between PD and HD in the NODM incidence was calculated with risk ratio (RR) and 95%CIs. In order to detect the potential source of heterogeneity, we performed sensitivity analysis by omitting one study at each turn. Publication bias was assessed by the Begg’s [25] and Egger’s test [26]. A two-tailed *P*-value of less than 0.05 was considered statistically significant, except where otherwise specified. All statistical analyses were performed using Stata 12.0 (Stata Corporation, College Station, TX, USA).

## Result

### Identification of eligible studies

**Fig. 1:**
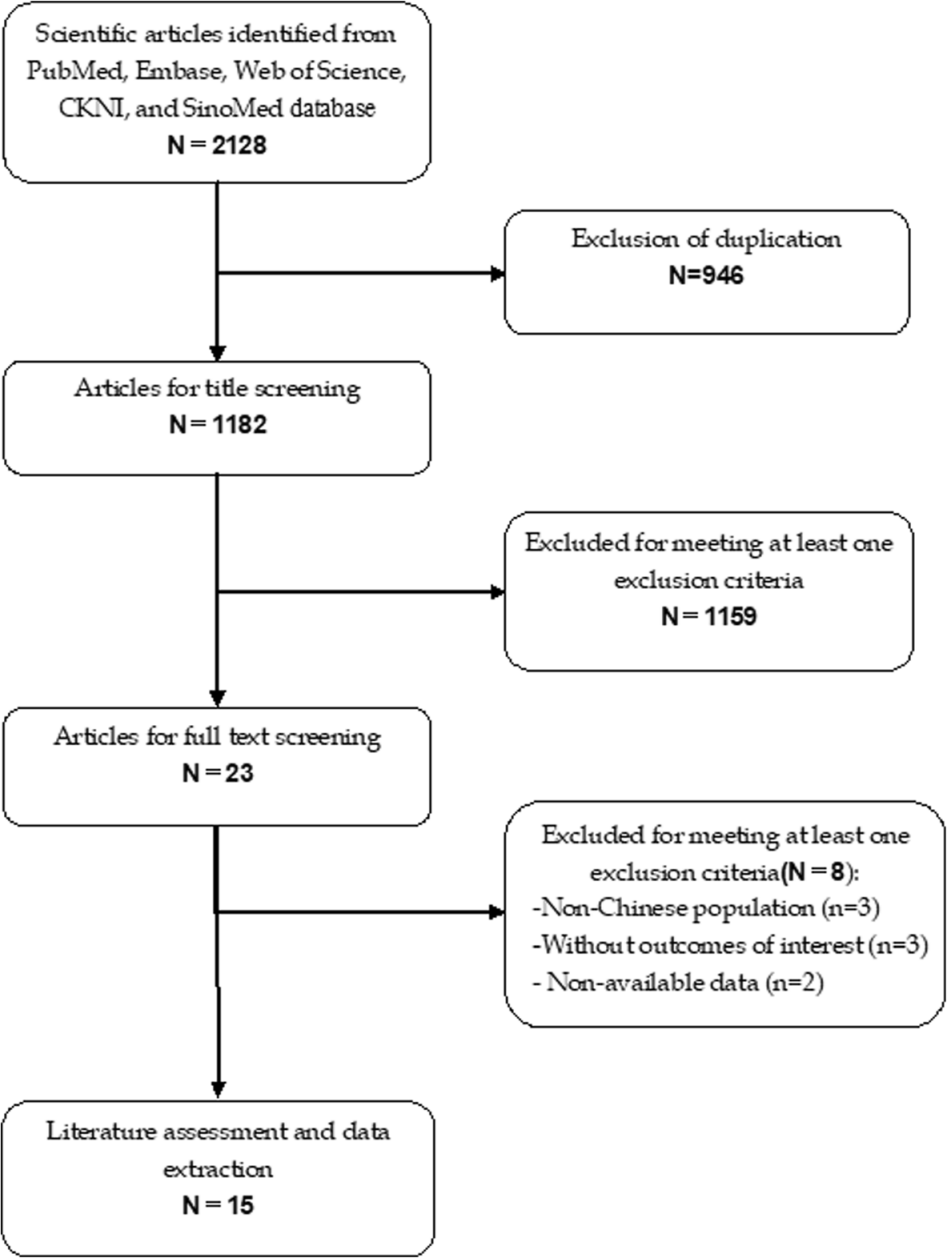
Eligibility of studies for inclusion in meta-analysis

The initial search yielded 2128 publications, of which 946 were excluded because of duplicate records. Then, 1182 publications were left for title/abstract review, and 1159 of them were removed because they were unrelated with our topics, or were reviews and case reports. The remaining 23 studies were reviewed for full-text information, and 8 of them were removed because three studies were not performed in Chinese population; three studies did not provide data of our interest; and two studies had non-available data for analysis. Finally, 15 studies met the inclusion criteria and were included in this meta-analysis [1, 11, 27–39] (Fig. 1).

### Study characteristics and quality assessment

The main characteristics of included studies were presented in Table 1. These studies were published between 2001 and 2019. All the studies were performed with retrospective or prospective cohort design. Eight studies were published in international English journals [1, 11, 27–32], whereas the remaining seven in Chinese journals [33–39]. The sample size varied greatly across the included studies, which ranged from 35 to 26,166. The mean ages of the patients were older than 40 years. Continuous ambulatory PD (CAPD) were applied in six of the included studies [11, 27, 32, 36–38], and the other studies used PD or HD. Based on the NOS for the quality assessment of cohort studies, these studies had a NOS score greater than 5 points, which indicated a high quality.

**Table 1:** Baseline characteristics of patients in the trials included in the meta-analysis

Study	Study design	Dialysis modality	No. of patients	Male/female	Age (mean ± SD, y)	Follow-up (months)	NOS score
Tien KJ [1]	Cohort	PD/HD	3346	1398/1948	NA	53.2	7
			22,820	10,784/12036	NA		
Szeto CC [11]	Cohort	CAPD	252	135/117	59 ± 13	45.4 ± 26.5	6
Yu XF [27]	Cohort	CAPD	145	60/85	62 ± 15	48	6
Wang IK [28]	Cohort	PD/HD	6177	2665/3512	51.0	49.44 ± 34.32	8
			6177	2726/3451	51.2	52.36 ± 37.2	7
Wu PP [29]	Cohort	PD/HD	2228	1096/1132	60.29 ± 16.43	64.2 ± 47.04	7
			8912	4384/4528	60.29 ± 16.43	73.68 ± 47.4	
Dong J [30]	Cohort	PD	32	9/23	61 ± 12.5	32.4 (12.9–60.8)	6
			580	256/324	55.2 ± 15.4		
Chou CY [31]	Cohort	PD/HD	2548	916/1632	50.2 ± 14.7	70	7
			10,192	3692/6500	50.3 ± 14.5		
Cheng SC [32]	Cohort	CAPD	14	5/9	46.4 ± 12.0	39.9 ± 28.3	6
			21	7/14	42.4 ± 9.4	60.5 ± 37.8	
Song ZP [33]	Cohort	PD	42	NA	60.2 ± 2.3	NA	6
			42	NA	60.2 ± 2.3		
Yang G [34]	Cohort	PD	40	NA	58.4 ± 4.7	NA	6
			42	NA	58.4 ± 4.7		
Ye SH [35]	Cohort	PD	44	24/20	60.14 ± 2.69	NA	6
			44	25/19	60.78 ± 2.98		
Peng XY [36]	Cohort	CAPD	138	83/55	57 (46–71)	NA	7
			69	38/31	56 (42–71)		
Xia P [37]	Cohort	CAPD/APD	442	228/214	58.4 ± 15.6	NA	6
			92	53/39	56.7 ± 16.0	NA	
Fei JY [38]	Cohort	CAPD	286	148/138	58 (13–85)	3–120	6
Li Y [39]	Cohort	PD	577	280/297	58.9 ± 15.5	NA	6

***Abbreviation***: *SD* standard deviation, *PD* Peritoneal dialysis, *HD* hemodialysis, *CAPD* continuous ambulatory PD, *APD*, automated PD

### Incidence of new-onset glucose metabolism disturbance

**Fig. 2:**
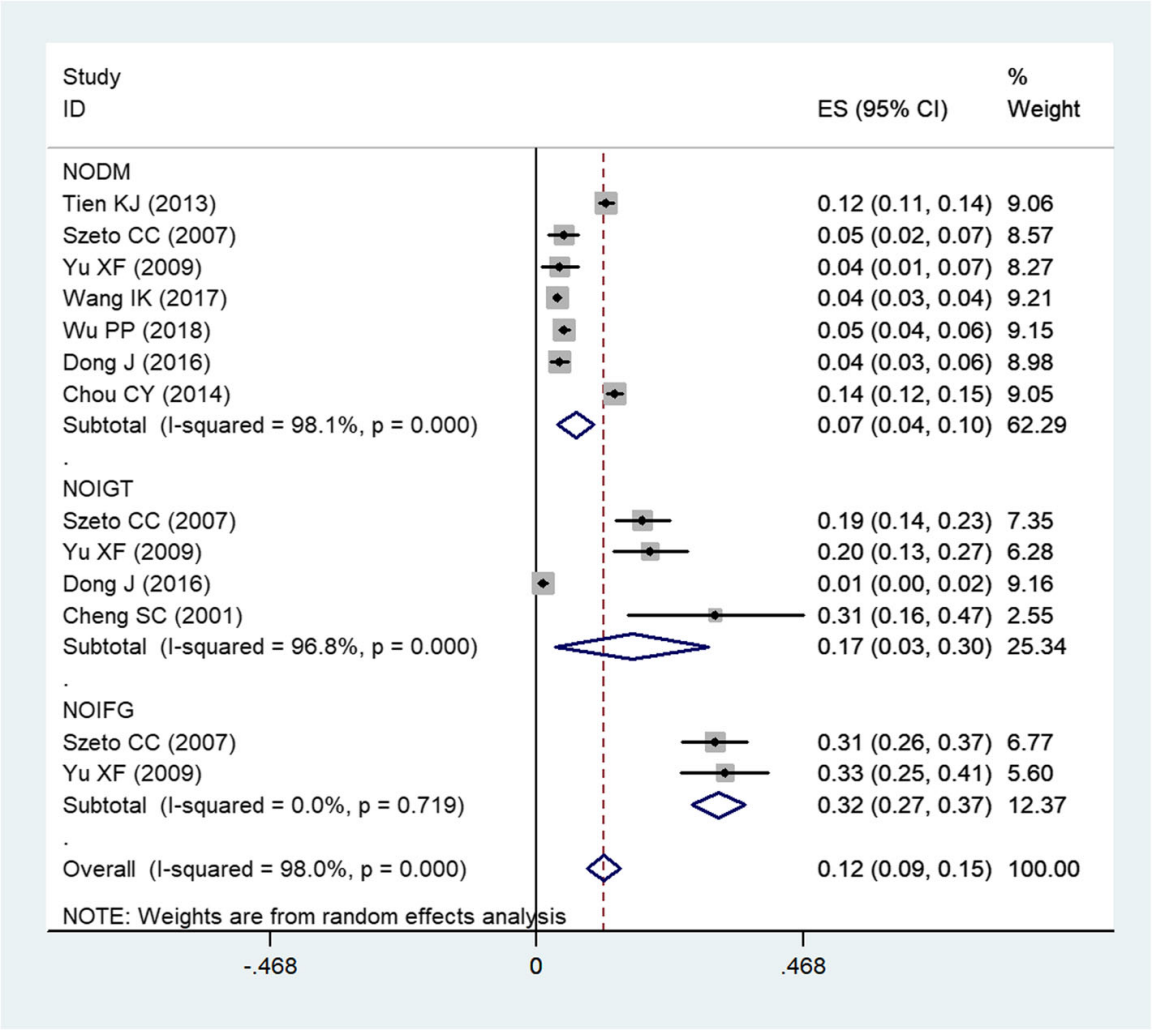
Forest plot showing the incidence of glucose metabolism disturbances in dialysis patients

Eight studies [1, 11, 27–32] reported the data of incidence of NOGMDs in dialysis patients. The incidence of NODM varied from 3.76 to 13.78% across the included studies. Pooled estimate showed that, 12% of patients developed NODM after dialysis (ES = 7, 95%CI: 4, 10%; *P* < 0.001) (Fig. 2). The test for heterogeneity was significant (I^2^ = 98.1%, *P* < 0.001), thus, we performed sensitivity analysis. When we excluded the trial with outlier [31], the pooled estimate changed a little (ES = 6, 95%CI: 3, 10%; *P* < 0.001). However, the heterogeneity was still present (I^2^ = 97.5%, *P* < 0.001). We further removed any single study, but the pooled result and heterogeneity did not alter substantially (data not shown).

The incidence of NOIGT in dialysis patients varied from 1.14 to 31.43% among the included studies. Pooled results indicated that, the incidence of NOIGT was 17% (ES = 17, 95%CI: 3, 30%; *P* < 0.001) in patients undergoing dialysis (Fig. 2).

The incidence of NOIFG was almost similar among the included two studies. Pooled results suggested that, 32% of patients developed NOIFG after they received dialysis (ES = 32, 95%CI: 3, 30%, *P* < 0.001) (Fig. 2).

### Mortality risk and NODM

**Fig. 3:**
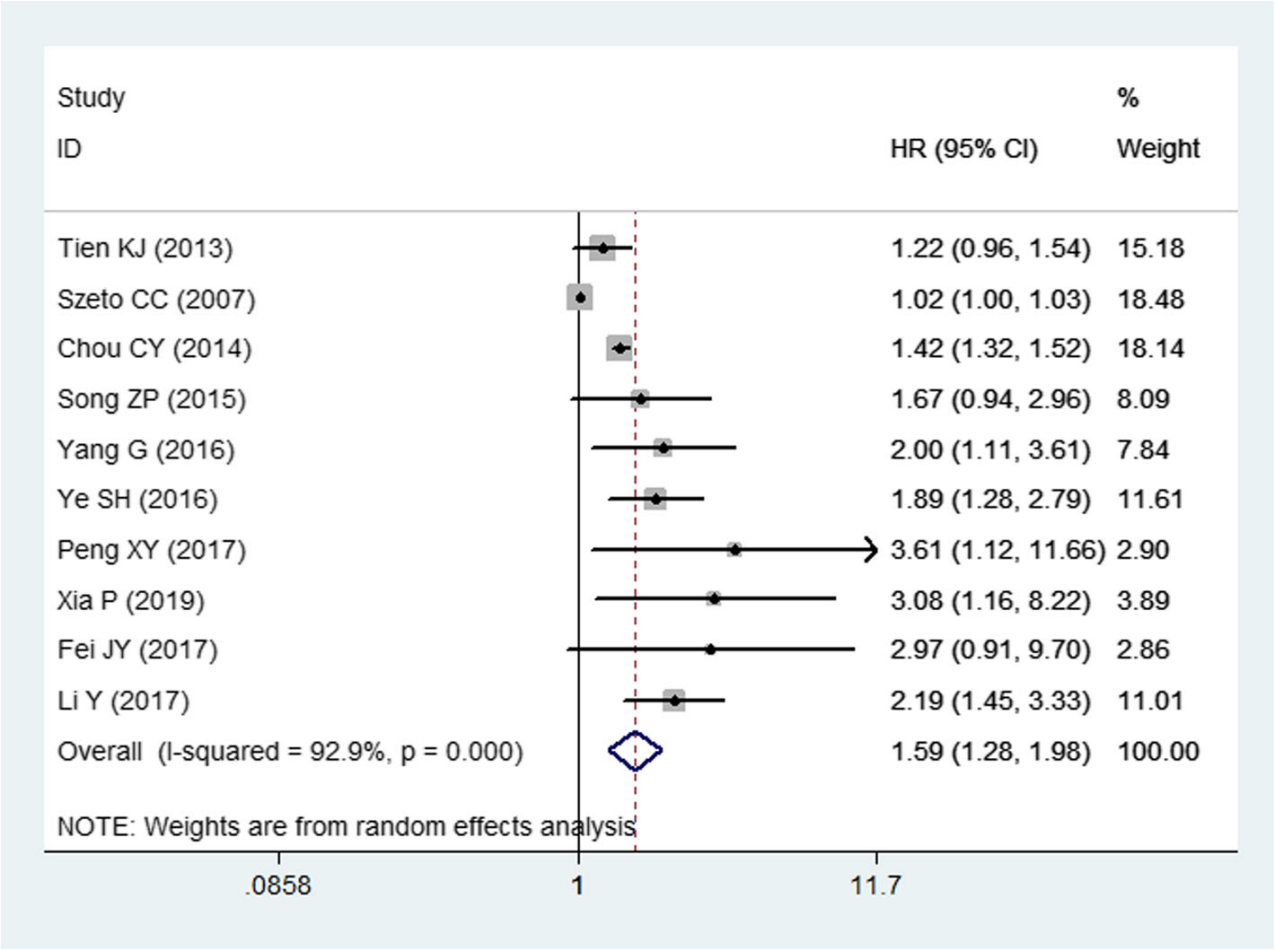
Forest plot showing the influence of NODM on the prognosis of PD patients

Ten studies [1, 11, 31, 33–39] reported the data of mortality in dialysis patients. Pooled the results showed that, PD patients who developed NODM had an increased risk of mortality than those without (HR = 1.59, 95%CI: 1.28, 1.98; *P* < 0.001) (Fig. 3). The test for heterogeneity was significant (I^2^ = 92.9%, *P* < 0.001), thus, we performed sensitivity analysis. When the trial with outlier [36], was removed, the overall estimate did not change substantially (HR = 1.55, 95%CI: 1.25, 1.93; *P* < 0.001), but the heterogeneity was still present (I^2^ = 93.4%, *P* < 0.001). When we excluded another trial with outlier [11], the summarized data altered slightly (HR = 1.67, 95%CI: 1.39, 2.00; *P* < 0.001), but the heterogeneity disappeared (I^2^ = 47.8%, *P* = 0.053). This indicated that the trial conducted by Szeto cc, et al. [11] contributed to the heterogeneity.

### Incidence of NODM between PD and HD

**Fig. 4:**
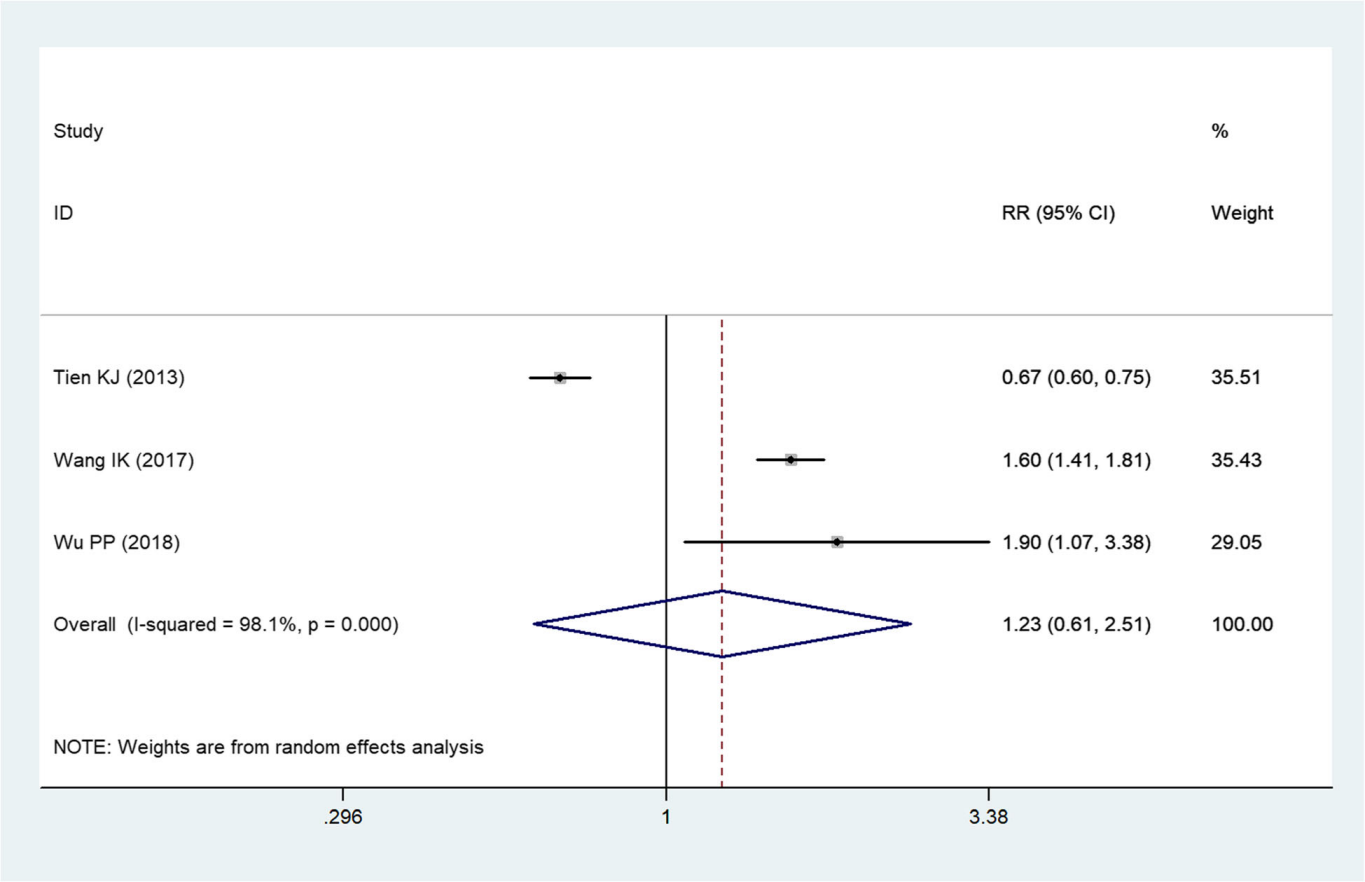
Forest plot showing the comparison between PD and HD in the incidence of NODM

Three studies [1, 28, 29] compared the incidence of NODM between PD and HD. Pooled results showed that, PD patients had a similar incidence of NODM with HD patients (RR = 1.23, 95%CI: 0.61, 2.51; *P* = 0.562) (Fig. 4).

### Publication bias

The assessment of publication bias showed that, there was no evidence of significant publication bias by the formal statistical tests (Egg test, *P* = 0.580; Begg test, *P* = 0.764).

## Discussion

The present meta-analysis included 15 studies to identify the risk of NOGMDs in Chinese patients undergoing dialysis, and the NODM incidence between PD and HD patients, as well as evaluate the impact of NODM on the survival outcome in PD patients. Our results showed that, the incidence of NODM in Chinese dialysis patients was 7% (95%CI: 4, 10%; *P* < 0.001). PD patients had similar risk with HD patients of developing NODM (RR = 1.23, 95%CI: 0.61, 2.51; *P* = 0.562). NODM increased the mortality rate in patients undergoing HD (HR = 1.59, 95%CI: 1.28, 1.98; *P* < 0.001). Our results were in consistent with the findings of the previous study conducted by Xue C, et al. [40] (Table 2).

**Table 2:** Outcome comparison between this and previous studies

	Xue C, et al.	Our study
Number of included studies	9	15
Total sample size	13,879	56,390
Population	Dialysis patients	Chinese dialysis patients
Incidence of NODM	8% (95%CI: 4, 12%)	12% (95%CI: 9, 15%)
Incidence of NOIGT	15% (95%CI: 3, 31%)	17% (95%CI: 4, 10%)
Incidence of NOIFG	32% (95%CI: 27, 37%)	32% (95%CI: 3, 30%)
Mortality rate in PD patients	HR = 1.06, 95%CI: 1.01, 1.44	HR = 1.59, 95%CI: 1.28, 1.98
Incidence of NODM between PD and HD	RR = 0.99, 95%CI: 0.69, 1.40	RR = 1.23, 95%CI: 0.61, 2.51

***Abbreviation***: *PD* Peritoneal dialysis, *HD* hemodialysis, *NODM* new-onset diabetes mellitus, *NOIFG* new-onset impaired fast glucose, *NOIGT* new-onset impaired glucose tolerance, *RR* risk ratio, *HR* hazard ratio, *CI* confidence intervals

The incidence of NODM in dialysis patients varied greatly across different counties and population. Salifu, MO, et al. [41] collected data from the United States Renal Data System (USRDS) from January 2000 to December 2001, and reported the incidence of NODM after HD was 20 per 1000 patient-year, and the prevalence was 7.6% during the 3 years of follow-up [41]. Whereas, in another study which was performed in China Taiwan, the rates of incidence and prevalence in dialysis patients with end-stage renal disease (ESRD) were higher than that in the previous study [1]. In that study, the authors examined the records of ESRD patients from the Taiwan National Health Insurance Research Database between 1999 and 2005 [1]. These patients who underwent dialysis were followed until death, transplant, dialysis withdrawal, or 31 December 2008. The cumulative incidence rate of NODM was 4% at 1 year and 21% at 9 years [1]. The incidence and prevalence of NODM at 10 year was 29 per 1000 patient-years and 12.8%, respectively [1]. Another study reported that the NODM prevalence in dialysis patients (PD and HD) at 6-year follow-up was 8.5% [13]. This was lower than that in the study of Tien KJ [1], which might be explained by the shorter follow-up period. Other factors may also have an impact on the risk of developing NODM. Woodward et al. [14] reported that, the immunosuppressant agents had great influence on the NODM. In their study, the incidence of NODM in patients who received cyclosporine and tacrolimus ranged from 18 to 30% after the transplant had been performed 2 years [14].

In the present study, we found the risk of developing NODM was not significant different between patients undergoing PD and HD. Our findings were in consistent with that of the previously published studies [1]. Tien KJ, et al. reported that, the incidence of NODM after initiation of dialysis in HD and PD group was 12.80 and 12.20%, respectively [1]. This difference was not significant even after adjustment. However, another study reported a significantly higher incidence of NODM in PD patients than in HD patients. In that study, the incidence of NODM in the two groups was 15.98 and 8.69 per 1000 patient-years, respectively (*P* < 0.001) [29]. Similarly, Woodward, RS, et al. [14] also reported different incidence of NODM among wait-listed transplanted renal allograft patients. The incremental incidence of NODM was 7.5% for PD patients and 8.2% for HD patients, respectively [14]. This difference might be biased by the confounders, including age, gender, pre-existing comorbidities (hypertension, cardiac disease, and cerebrovascular disease), which had not been adjusted in the data analysis.

Regarding the survival outcome, our study found that, dialysis patients with NODM had a significantly higher mortality rate than those without NODM. Our results were in consistent with the findings in the previous studies. Chou CY, et al. [31] examined the data in Taiwan Renal Registry Database from 1997 to 2005in 2548 PD patients and 10,192 HD patients who had no DM on the initiation of dialysis [31]. Data analysis using the propensity score matching method suggested that, NODM was associated with a significantly increased risk of mortality (HR = 1.42, 95%CI: 1.32, 1.52; *P* < 0.001) [31]. Similarly, Salifu, MO et al. [2] found that the mortality rate at the end of 3-year follow-up was significantly higher in NODM patients with HD (49.2%), second highest in patients with pre-existing DM (50.6%) than those without DM (41%) [2]. Tien, KJ, et al. [1] found similar results with the above findings, in which the survival rate was significantly highest in those without DM, moderate in those with NODM, and lowest in those with pre-existing DM. Compared with those without DM, pre-existing DM increased the death risk by 80% (HR = 1.81, 95%CI: 1.75, 1.87); whereas NODM increased the death risk by 10% (HR = 1.10, 95%CI: 1.03, 1.17) [1]. An interesting finding in the Kaplan-Meier survival plot showed that, the survival curves of patients with NODM and without DM began to diverge at 3 years after initiation of dialysis therapy [1]. The authors suggested that, this might reflect the cumulative or delayed damage caused by the increased glucose level [1].

The use of osmotic agents could have an impact on the reduction of GMDs. Icodextrin, derived from cornstarch, is a glucose polymer, which is a commonly used glucose-sparing solution [41]. By using the icodextrin, the ultrafiltration is enhanced and uncontrolled fluid overload is mitigated [42]. This solution has metabolic effects on the glycemic and dyslipidemia control [43–45]. The use of icodextrin may improve the technique and survival in PD patients [46–48]. Wang IK, et al. collected data from Taiwan health insurance database from 2000 to 2010 to assess the effects of icodextrin in reducing the risk of NODM in PD patients [28]. Their results suggested that, among PD patients, the incidence of NODM was significantly lower in icodextrin users than n nonusers (6.22 VS 12.1 per 1000 person-years), with an adjusted HR of 0.66 (95%CI: 0.50, 0.88) [28]. The authors concluded that, icodextrin was effective in reducing the risk of NODM in PD patients.

There were several potential limitations in this study. First, significant heterogeneity was identified across the included studies. Although sensitivity analysis was performed to explore the potential sources, no valuable information was obtained from the analysis results. However, there were various factors across the included studies, including definition of DM, severity of the ESRD, age of initial dialysis, baseline comorbidity, and the duration of follow-up. These factors might account for the heterogeneity and had impact on the final results. Second, all the included studies were retrospective or prospective cohort studies. Although the cohort studies can reflect the “real-world” and further support the conclusion, cohort data are of course highly prone to selection bias. Third, it would be interesting to compare the influence between pre-existing and new-onset DM on the prognosis of dialysis patients; however, due to the limited data, these data analysis was not performed. Fourth, there were four studies that were derived from the analysis of Taiwan National Health Care Insurance Database with different time periods. However, some of them had the overlap data duration the same period. We have tried to contact the corresponding authors with requests for the original data. However, none of them responded to our request. The overlapped data would result in bias in the data analysis and influence the effect size. Thus, the physicians should interpret our results with caution.

## Conclusion

The present study confirmed the risk of developing NODM in Chinese patients with dialysis; however, the dialysis modality did not have any significant impact on the incidence of NODM. The development of NODM increased the mortality risk in patients undergoing PD. Thus, physicians should pay attention to the plasma glucose level in patients undergoing dialysis.

## Data Availability

All data generated and analysed during this study are included in this published article.

## Abbreviations

CAPD: Continuous ambulatory PD
CI: Confidence intervals
CKD: Chronic kidney disease
DM: Diabetes mellitus
ES: Estimate
GMD: Glucose metabolism disturbances
HR: Hazard ratio
IFG: Impaired fast glucose
IGT: Impaired glucose tolerance
NODM: New-onset diabetes mellitus
PD: Peritoneal dialysis
RR: Risk ratio

## References

[1] Tien KJ, Lin ZZ, Chio CC, Wang JJ, Chu CC, Sun YM, Kan WC, Chien CC (2013). Epidemiology and mortality of new-onset diabetes after dialysis: Taiwan national cohort study. Diabetes Care.

[2] Salifu MO, Abbott KC, Aytug S, Hayat A, Haria DM, Shah S, Friedman EA, Delano BG, McFarlane SI, Hurst FP (2010). New-onset diabetes after hemodialysis initiation: impact on survival. Am J Nephrol.

[3] Lin-Tan DT, Lin JL, Wang LH, Wang LM, Huang LM, Liu L, Huang JY, Huang YL (2007). Fasting glucose levels in predicting 1-year all-cause mortality in patients who do not have diabetes and are on maintenance hemodialysis. J Am Soc Nephrol.

[4] Tabak AG, Herder C, Rathmann W, Brunner EJ, Kivimaki M (2012). Prediabetes: a high-risk state for diabetes development. Lancet (London, Engl).

[5] Kanaya AM, Herrington D, Vittinghoff E, Lin F, Bittner V, Cauley JA, Hulley S, Barrett-Connor E (2005). Impaired fasting glucose and cardiovascular outcomes in postmenopausal women with coronary artery disease. Ann Intern Med.

[6] System. USRD: USRDS 2014 Annual Data Report: atlas of chronic kidney and end-stage renal disease in the United States Bethesda, MD: National Institute of Health, National Institute of Diabetes and Digestive and Kidney Disease*s* 2014.

[7] DeFronzo RA, Alvestrand A, Smith D, Hendler R, Hendler E, Wahren J (1981). Insulin resistance in uremia. J Clin Invest.

[8] Kobayashi S, Maesato K, Moriya H, Ohtake T, Ikeda T (2005). Insulin resistance in patients with chronic kidney disease. Am J Kidney Dis.

[9] Landau M, Kurella-Tamura M, Shlipak MG, Kanaya A, Strotmeyer E, Koster A, Satterfield S, Simsonick EM, Goodpaster B, Newman AB (2011). Correlates of insulin resistance in older individuals with and without kidney disease. Nephrol Dialysis Transplant.

[10] Kobayashi S, Maejima S, Ikeda T, Nagase M (2000). Impact of dialysis therapy on insulin resistance in end-stage renal disease: comparison of haemodialysis and continuous ambulatory peritoneal dialysis. Nephrol Dialysis Transplant.

[11] Szeto CC, Chow KM, Kwan BC, Chung KY, Leung CB, Li PK (2007). New-onset hyperglycemia in nondiabetic chinese patients started on peritoneal dialysis. Am J Kidney Dis.

[12] Hoffmann F, Haastert B, Koch M, Giani G, Glaeske G, Icks A (2011). The effect of diabetes on incidence and mortality in end-stage renal disease in Germany. Nephrol Dialysis Transplant.

[13] Lok CE, Oliver MJ, Rothwell DM, Hux JE (2004). The growing volume of diabetes-related dialysis: a population based study. Nephrol Dialysis Transplant.

[14] Woodward RS, Schnitzler MA, Baty J, Lowell JA, Lopez-Rocafort L, Haider S, Woodworth TG, Brennan DC (2003). Incidence and cost of new onset diabetes mellitus among U.S. wait-listed and transplanted renal allograft recipients. Am J Transplant Off J Am Soc Transplant Am Soc Transplant Surg.

[15] Verduijn M, Grootendorst DC, Dekker FW, Jager KJ, le Cessie S (2011). The analysis of competing events like cause-specific mortality--beware of the Kaplan-Meier method. Nephrol Dialysis Transplant.

[16] Chou CY, Wang IK, Liu JH, Lin HH, Wang SM, Huang CC (2010). Comparing survival between peritoneal dialysis and hemodialysis treatment in ESRD patients with chronic hepatitis C infection. Peritoneal Dialysis Int.

[17] Weinhandl ED, Foley RN, Gilbertson DT, Arneson TJ, Snyder JJ, Collins AJ (2010). Propensity-matched mortality comparison of incident hemodialysis and peritoneal dialysis patients. J Am Soc Nephrol.

[18] Diaz-Buxo JA, Lowrie EG, Lew NL, Zhang H, Lazarus JM (2000). Quality-of-life evaluation using short form 36: comparison in hemodialysis and peritoneal dialysis patients. Am J Kidney Dis.

[19] Yang WC, Hwang SJ. Incidence, prevalence and mortality trends of dialysis end-stage renal disease in Taiwan from 1990 To 2001: the impact of national health insurance. Nephrol dialysis Transplant. 23(12):3977–82. 10.1093/ndt/gfn406 18628366

[20] Higgins JP, Thompson SG, Deeks JJ, Altman DG (2003). Measuring inconsistency in meta-analyses. BMJ.

[21] Moher D, Liberati A, Tetzlaff J, Altman DG (2009). Preferred reporting items for systematic reviews and meta-analyses: the PRISMA statement. BMJ.

[22] Wells G, Shea B, O’connell D, Peterson J, Welch V: The Newcastle-Ottawa Scale (NOS) for assessing the quality of nonrandomized studies in meta-analyses**.** . *3rd Symposium on Systematic Reviews: Beyond the Basics* 2000:3–5.

[23] DerSimonian R, Laird N (1986). Meta-analysis in clinical trials. Control Clin Trials.

[24] Mantel N, Haenszel W (1959). Statistical aspects of the analysis of data from retrospective studies of disease. J Natl Cancer Inst.

[25] Begg CB, Mazumdar M (1994). Operating characteristics of a rank correlation test for publication bias. Biometrics.

[26] Egger M, Davey Smith G, Schneider M, Minder C (1997). Bias in meta-analysis detected by a simple, graphical test. BMJ.

[27] Yu XF, Zhou YF, Feng L, Zhang DL, Liu WH (2009). Influence of peritoneal transfer status on fasting blood glucose in non-diabetic nephropathy patients on continuous ambulatory peritoneal dialysis. Chin Med J.

[28] Wang IK, Lin CL, Chen HC, Lin SY, Chang CT, Yen TH, Sung FC (2018). Risk of new-onset diabetes in end-stage renal disease patients undergoing dialysis: analysis from registry data of Taiwan. Nephrol Dialysis Transplant.

[29] Wu PP, Kor CT, Hsieh MC, Hsieh YP. Association between End-Stage Renal Disease and Incident Diabetes Mellitus-A Nationwide Population-Based Cohort Study. J Clin Med. 2018:**7**(10). 10.3390/jcm7100343 PMC6210467 30314341

[30] Dong J, Yang ZK, Chen Y (2016). Older age, higher body mass index and inflammation increase the risk for new-onset diabetes and impaired glucose tolerance in patients on peritoneal Dialysis. Peritoneal Dialysis Int.

[31] Chou CY, Liang CC, Kuo HL, Chang CT, Liu JH, Lin HH, Wang IK, Yang YF, Huang CC (2014). Comparing risk of new onset diabetes mellitus in chronic kidney disease patients receiving peritoneal dialysis and hemodialysis using propensity score matching. PLoS One.

[32] Cheng SC, Chu TS, Huang KY, Chen YM, Chang WK, Tsai TJ, Wu KD (2001). Association of hypertriglyceridemia and insulin resistance in uremic patients undergoing CAPD. Peritoneal Dialysis Int.

[33] Song ZP (2015). Analysis of clinical characteristics and prognosis of diabetic nephropathy patients on peritoneal dialysis. Contemp Med Forum.

[34] Yang G, Li XY, Fang WD (2016). Analysis of clinical features and prognosis of patients with diabetic nephropathy on peritoneal dialysis. Contemp Med.

[35] Ye SH (2016). Clinical features and prognosis of patients with diabetic nephropathy on peritoneal dialysis. China Health Care Nutr.

[36] Peng XY, Wang HY, Li Y, Wang Y, Zhou ZJ, Ma Y, Liu BY, Yang W, Cui Y, Li XM (2017). Survival analysis in automated peritoneal dialysis patients. Chin J Nephrol.

[37] Xia P, Wang HY, Li Y, Wang Y, Tian DL, Zhou ZJ, Zhen H, Li Y, Liu BY, Qin Y (2019). Long-term prognosis of end stage diabetic kidney disease patients treated with automated peritoneal dialysis. Chin J Blood Purif.

[38] Fei JY, Qiu XH (2017). Clinical analysis of five-year survival rate in patients with peritoneal dialysis. Zhejiang Clin Med J.

[39] Li Y, Wang HY, Wang Y, Zhou ZJ, Liu BY, Yang W, Cui Y, Li XM, Chen LM (2017). Long-tem surviVal analysis of the elderly peritoneal dialysis patients. Chin J Nephrol.

[40] Xue C, Gu YY, Cui CJ, Zhou CC, Wang XD, Ruan MN, Huang LX, Chen SX, Yang B, Chen XJ *et al*. New-onset glucose disorders in peritoneal dialysis patients: a meta-analysis and systematic review. *Nephrology, dialysis, transplantation : official publication of the European Dialysis and Transplant Association - European Renal Association* 2019. 10.1093/ndt/gfz116 31236586

[41] Frampton JE, Plosker GL (2003). Icodextrin: a review of its use in peritoneal dialysis. Drugs.

[42] Cho Y, Johnson DW, Badve S, Craig JC, Strippoli GF, Wiggins KJ (2013). Impact of icodextrin on clinical outcomes in peritoneal dialysis: a systematic review of randomized controlled trials. Nephrol Dialysis Transplant.

[43] Bredie SJ, Bosch FH, Demacker PN, Stalenhoef AF, van Leusen R (2001). Effects of peritoneal dialysis with an overnight icodextrin dwell on parameters of glucose and lipid metabolism. Peritoneal Dialysis Int.

[44] Li PK, Culleton BF, Ariza A, Do JY, Johnson DW, Sanabria M, Shockley TR, Story K, Vatazin A, Verrelli M (2013). Randomized, controlled trial of glucose-sparing peritoneal dialysis in diabetic patients. J Am Soc Nephrol.

[45] Marshall J, Jennings P, Scott A, Fluck RJ, McIntyre CW (2003). Glycemic control in diabetic CAPD patients assessed by continuous glucose monitoring system (CGMS). Kidney Int.

[46] Han SH, Ahn SV, Yun JY, Tranaeus A, Han DS (2012). Effects of icodextrin on patient survival and technique success in patients undergoing peritoneal dialysis. Nephrol Dialysis Transplant.

[47] Wang IK, Li YF, Chen JH, Liang CC, Liu YL, Lin HH, Chang CT, Tsai WC, Yen TH, Huang CC (2015). Icodextrin decreases technique failure and improves patient survival in peritoneal dialysis patients. Nephrology (Carlton, Vic).

[48] Wang IK, Lu CY, Muo CH, Chang CT, Yen TH, Huang CC, Li TC, Sung FC (2016). Analysis of technique and patient survival over time in patients undergoing peritoneal dialysis. Int Urol Nephrol.

